# Spatial Analysis of Prediabetes and Associated Risk Factor Prevalence Among Late Adolescents in San Luis Potosí, México

**DOI:** 10.7759/cureus.72568

**Published:** 2024-10-28

**Authors:** Patricia E Cossío-Torres, Rogelio Santana-Arias, Margarita Teran-Garcia, Juan M Vargas-Morales, Marisol Vidal-Batres, Carlos A González-Cortés, Mariela Vega-Cárdenas, Celia Aradillas-García

**Affiliations:** 1 Faculty of Medicine, Universidad Autónoma de San Luis Potosí, San Luis Potosí, MEX; 2 Faculty of Engineering, Universidad Autónoma de San Luis Potosi, San Luis Potosí, MEX; 3 Division of Nutritional Sciences, University of Illinois Urbana-Champaign, Champaign, USA; 4 Faculty of Chemical Sciences, Universidad Autónoma de San Luis Potosí, San Luis Potosí, MEX; 5 School of Human Nutrition, McGill University, Montreal, CAN; 6 Coordination for the Innovation and Application of Science and Technology, Universidad Autónoma de San Luis Potosí, San Luis Potosí, MEX

**Keywords:** late adolescence, overweight and obesity, prediabetes prevalence, risk factors, spatial distribution

## Abstract

Introduction

The prevalence of prediabetes is increasing worldwide. However, the determinants that contribute to its onset in young individuals remain poorly understood. An essential aspect of directing control and preventive initiatives is comprehending the geographical distribution of these disorders and pinpointing regions with a high prevalence.

Objective

The objective of this study was to determine the spatial distribution of prediabetes and associated risk factor prevalence among late adolescents in the metropolitan area of San Luis Potosí (MASLP), México, during the years 2008, 2009, and 2010.

Methods

This was a cross-sectional study that included 15,672 participants between the ages of 18 and 21 years. We made a cartographic overlay of the body mass index (BMI), systolic blood pressure (BP), diastolic BP, and impaired fasting glucose (IFG) using a 2010 marginalization index.

Results

The prevalence of prediabetes was 5.5%, whereas the prevalence of overweight and obesity remained stable for three years. However, in 2010, the prevalence of both diastolic BP and prediabetes increased. Spatial analysis revealed that the urban basic geostatistical area (Area Geo-Estadística Básica (AGEB)), with 11-20 participants with prediabetes, was mainly concentrated in the areas of medium and low marginalization throughout all years.

Conclusion

We found a yearly increase in prediabetes prevalence and increased diastolic BP. Prediabetes varies across the region. MASLP has statistically detected significant high hot spots in prediabetes. The findings of this study are valuable for directing resource distribution and highlighting intervention programs that target modifiable prediabetes predictors. To improve health coverage, it is necessary to consider different local realities.

## Introduction

Prediabetes is a state characterized by altered glucose metabolism associated with a higher risk of developing type 2 diabetes (T2D). Impaired fasting glucose (IFG) levels between 100 and 125 mg/Dl, impaired glucose tolerance (IGT) levels between 140 and 199 mg/dL after two hours of an oral glucose test, and glycosylated hemoglobin (HbA1c) levels between 5.7% and 6.4% are commonly used to define prediabetes, based on the American Diabetes Association diagnosis criteria [1]. Otherwise, modifiable risk factors, including obesity and altered blood pressure, are associated with an increased risk of developing prediabetes and diabetes [2,3]. The identification of modifiable and nonmodifiable factors that are associated with prediabetes could be helpful for developing effective prevention strategies.

The distinct definitions of prediabetes have led to inconsistent prevalence estimates and complicated comparisons across time and populations. According to the 2021 International Diabetes Federation (IDF) statistics, 10.6% of the adult population has IGT, and 6.2% has IFG [4]. Several studies portray this situation in Mexico. Using the gathered capillary blood samples, it has been reported that there is a 14.3% prevalence of prediabetes in adolescents (<20 years old) and 21.8% in adults (>20 years old) [5]. Another study reported a 9.8% prevalence of prediabetes in the 20-30-year-old age group in the rural Mexican population, also based on glucose levels obtained from capillary blood samples [6]. On the other hand, a population-based study from 2010 to 2012 revealed that 11% of indigenous individuals and 18% of nonindigenous individuals had prediabetes [7]. According to this report, 13.4% of these individuals received a diagnosis based on both IGT and IFG, 52.4% on IFG alone, and 34.2% solely on IGT. The first serial cross-sectional analysis of the National Health and Nutrition Survey (ENSANUT) in Mexico (2016-2022) reported a prevalence of prediabetes (either IFG or high HbA1c) of 20.9% in 2022 in adults aged 20-60 years or older [8]. The above shows that in Mexico, there is scarce information about the prevalence of prediabetes in late adolescence and youth.

Late adolescence is the phase of life between childhood and adulthood, from ages 18 to 21 years old [9]. During this phase, academic performance, family relations, social groups, and resting time influence the lifestyle behaviors of adolescents that typically persists into adulthood, making them a vulnerable group [10]. Few studies have evaluated the prevalence of prediabetes among the Mexican young population. Muñoz Cano et al. reported in 2011 that newly enrolled university students had a prevalence of 10% prediabetes and 1.5% T2D [11]. Another study using IFG or IGT found a 14.6% prevalence of prediabetes in the population aged 18-30 years [12]. It is important to monitor the prevalence of glucose dysregulation in young adults in order to guide prevention strategies and identify subgroups at higher risk for early-onset T2D.

Several studies highlight the importance of diabetes research at a local scale; as existing sociodemographic variables relate to the prevalence of diabetes [13,14]. Because these have long been recognized as essential components of the epidemiological sciences, understanding their impact on health is a key element of public health and epidemiologic research. Currently, researchers are employing a variety of tools and paradigms to analyze spatial and geographic data related to health and risk factors. Spatial and geographic data analysis is a promising venue for epidemiological and public health research because it allows for the detection of different risk factors in the prevalence of diabetes and can, therefore, assist in its monitoring at a local and state level [14]. Not much is known about how the patient's environment's spatial prevalence affects prediabetes prevalence. Data suggests that prediabetes risk factors vary according to exposure differences to environmental risk factors and ethnic stratification, both of which constitute geographically and genetically susceptible groups [15]. Furthermore, information regarding the risk and socioeconomic factors of groups associated with the prevalence of prediabetes and their spatial distribution may help legislators consider the implementation of programs aimed at diabetes prevention and the development of community and clinical services.

Due to the high prevalence of prediabetes and associated risk factors currently experienced worldwide, it is critical to monitor individuals living with prediabetes to better understand the scope of the problem. Also, it is imperative to evaluate individuals in late adolescence to identify those at risk and prevent the natural evolution of this disease. Thus, the purpose of this research was to (1) identify the prevalence of prediabetes and associated risk factors in late adolescence, (2) assess the spatial distribution of these variables in a major metropolitan area, and (3) monitor the conduct of these variables in a span of three years (i.e., 2008-2010).

## Materials and methods

This was a retrospective study carried out from 2017 to 2021 in the capital of San Luis Potosí (San Luis Potosí City), located 363 kilometers northeast of Mexico City (22° 09´ 04” N, 100° 58´ 34” W). It is at an altitude of 1,860 meters above sea level, and its climate is predominantly dry to semi-dry.

Data collection

Data used in the present study was collected from the macroproject UP AMIGOS (Autonomous University of San Luis Potosi and Illinois University: a Multidisciplinary Investigation on Genetics, Obesity, and, Social Environment). The project was registered as Detection of Lifestyle, Phenotype, and Genotype, Risk for Chronic-Metabolic Diseases: Type 2 Diabetes, Obesity, and Hypertension in Young Adults of San Luis Potosi with the Bioethics and Research Committee of the state of San Luis Potosí and was given approval in 2008 (SLP/012-2008), 2009 (SLP/012-2009), and 2010 (SLP/012-2010) before data collection. Annual data of newly enrolled undergraduate students at a public university in San Luis Potosí, Mexico, was collected in the project. Eligibility required participants to be aged 18-21 years and be residents of San Luis Potosí, Mexico. Students with a documented medical history of diabetes and/or cardiovascular diseases and pregnant women were not included. Over 10,000 individuals were recruited and screened for this multidisciplinary investigation [16]. All applicants to the Autonomous University of San Luis Potosí were evaluated for anthropometric, biochemical, clinical, and dietary indicators prior to the entrance exam.

All the participants provided written consent for the use of the data for research purposes and their public availability. Data from 15,672 participants aged 18-21 years of age were collected from February to July each year in 2008, 2009, and 2010. Each participant underwent a clinical examination consisting of (a) anthropometric measurements (weight and height), (b) clinical measurements (BP), (c) blood sample measurements (with 12 hours of fasting to determine biochemical parameters), and (d) family history of diabetes (FHD) and/or cardiovascular diseases (FHCVD). 

*Anthropometric and Clinical Measurements* 

Weight and height were measured without shoes and with light clothing. Weight was measured with a calibrated scale to the nearest 0.1 kg (Torino, Mexico). Height was measured using a stadiometer to the nearest 0.5 cm. Additionally, we considered the following categories of the World Health Organization (WHO)-classified body mass index (BMI) according to Quetelet index (kg/m^2^): underweight (18.5 kg/m^2^), normal weight (18.5-24.9 kg/m^2^), overweight (25.0-29.9 kg/m^2^), and obesity (30 kg/m^2^). BMI was considered increased when this was within the categories of overweight and obesity [17].

BP was taken on the dominant arm in the seated position using appropriately sized Welch Allyn cuffs. Trained healthcare providers measured both systolic BP (SBP) and diastolic BP (DBP), which were measured and categorized according to the 2017 guidelines for the prevention, detection, evaluation, and management of high BP in adults by the American College of Cardiology/American Heart Association Task Force [18]. SBP was categorized as (1) normal (<120 mmHg), (2) increased (120-129 mmHg), and (3) hypertension (>130 mmHg). Diastolic BP was categorized as (1) normal (<80 mmHg), and (2) hypertension (>80 mmHg). Increased SBP and DBP were defined when BP values were within the categories of increased BP or hypertension [19].

Blood samples were collected after 12 hours of fasting. Fasting blood glucose (FBG) was determined according to the glucose oxidase peroxidase method (Alcyon 300; Abbott Laboratories, Chicago, Illinois, United States). IFG ≥100-125 mg/Dl was defined as prediabetes [1].

Spatial distribution of variables

The present study started in 2017 with the georeferencing of participants according to their address. We geocoded all addresses to obtain geographic coordinates and then performed a spatial analysis of prediabetes and other cardiometabolic factors in the late adolescent population aged 18-21 years. In 2018, we prepared maps and tables (descriptive statistics of the data), including the frequencies of the variables by year and by sex. This process concluded in 2021.

We used the participants’ home addresses at the time they underwent the medical examinations from the UP AMIGOS project [16] to integrate their personal data and risk factors into a spatial database using Google Maps and Google Earth (Google LLC, Mountain View, California, United States). We assigned the latitude and longitude at the centroid of the home address, thereby localizing their home's street, apartment complex, avenue, and house number. When a participant provided only the name of a street or apartment complex, we substituted the centroid of a polygon spanning the apartment complex or the centroid of their street. Latitude and longitudinal coordinates were then converted into a point shapefile document using ArcGIS 10.7 software (Environmental Systems Research Institute, Inc., Redlands, California, United States), where each point represents the address of each participant. Subsequently, these files were cross-referenced with a shapefile document of the base geostatistical area (Area Geo-Estadística Básica (AGEB)), which constitutes the metropolitan area of San Luis Potosi (MASLP). With this, the total number of points that each AGEB has was counted to identify the total number of participants at risk with the following altered parameters: BMI, SBP, DBP, and glucose.

Lastly, we created a cartographic overlay of the variables using a 2010 marginalization index, which the National Population Council had prepared based on the 2010 census [20]. The marginalization index is a tool that allows us to differentiate between urban populations in Mexico. The marginalization index is calculated based on the percentage of the population aged 6-14 years that does not attend school (i.e., elementary/middle school), percentage of the population 15 years and older without basic/primary education, percentage of the population without access to public health services, percentage of private home occupants without drainage or toilets, percentage of private home occupants without electricity, percentage of private home occupants without proper piping systems, percentage of private home occupants with dirt floors, percentage of private home occupants with overcrowding, percentage of private home occupants without refrigerators, percentage of private home occupants without internet access, and percentage of private home occupants without cellular phones. Lastly, to better interpret the postulated marginal index, we implemented a 5-scale rating that can be categorized as very low, low, medium, high, and very high marginalization index.

Statistical analysis

Descriptive statistics were used in all variables, including the mean, the standard deviation (SD) in the continuous variables, and the proportions for categorical variables. The Kolmogorov-Smirnov test verified the normal distribution in the continuous variables. We performed a Student's t-test to assess the difference between male and female individuals in the continuous variables. Additionally, we conducted a chi-square analysis to assess the association between the categorical variables, followed by a statistical analysis. p-values <0.05 values were considered statistically significant. We performed statistical analyses using IBM SPSS Statistics for Windows, Version 23.0 (Released 2015; IBM Corp., Armonk, New York, United States).

## Results

A total of 15,672 participants were included in this study with a mean age of 18.6 years (±0.9, ranging from 18 to 21 years). They were 7634 (48.7%) men and 8038 (51.3%) women. The mean and SD for age, height, weight, BMI, SBP, DBP, and glucose levels are shown in Table 1. We found significant gender differences in SDP, DBP, BMI, and glucose levels, which were higher in male participants.

**Table 1:** General characteristics of the study population BMI: body mass index; SBP: systolic blood pressure; DBP: diastolic blood pressure. Student's t test was performed.

Variable	Total (n=15672), mean±SD	Male (n=7634), mean±SD	Female (n=8038), mean±SD	Significance		
				t	dif	p-value
Age (years)	18.6±0.9	18.74±0.9	18.59±0.8	10.4	0.15	<0.001
Height (m)	1.6±0.0	1.71±0.0	1.59±0.0	125.0	0.12	<0.001
Weight (kg)	64.8±14.9	71.03±14.9	59.02±12.3	54.9	12.01	<0.001
BMI (kg/m^2^)	23.7±4.4	24.11±4.5	23.35±4.4	10.6	0.75	<0.001
SBP (mmHg)	110.1±10.3	113.82±9.9	106.56±9.4	46.7	7.2	<0.001
DBP (mmHg)	71.9±8.0	74.32±7.5	69.77±7.8	37.0	4.5	<0.001
Glucose (mg/dL)	86.8±12.0	88.29±10.4	85.39±13.2	15.1	2.8	<0.001

Table 2 shows the prevalence of the risk factors analyzed in the present study. The most prevalent factors were increased SBP and DBP, followed by overweight and obesity. The sample's overall prediabetes prevalence was 5.5%.

**Table 2:** Prediabetes and associated risk factor prevalence SBP: systolic blood pressure; DBP: diastolic blood pressure

Risk factor	Total (n=15672), n (%)	Male (n=7634), n (%)	Female (n=8038), n (%)
Prevalence of prediabetes	862 (5.5)	565 (7.4)	297 (3.7)
Prevalence of increased DBP	4325 (27.6)	3122 (40.9)	1198 (14.9)
Prevalence of increased SBP	5814 (37.1)	3657 (47.9)	2154 (26.8)
Prevalence of overweight	3479 (22.2)	1847 (24.2)	1624 (20.2)
Prevalence of obesity	1473 (9.4)	801 (10.5)	675 (8.4)

Table 3 shows that among the altered risk factors, the odds of prevalent prediabetes were statistically significant, suggesting that altered BMI, SBP, and DBP are useful in predicting prediabetes in late adolescents.

**Table 3:** Odds ratio of prediabetes and altered risk factor The bilateral asymptotic significance of the chi-square test was less than 0.001 for all variables except SBP. ^a^ Person's Chi-square value, ^b^ Likelihood ratio value, 1 degree of freedom.

Risk factor		Normal fasting glucose, n (%)	Prediabetes, n (%)	Χ^2^		OR	CI	P-value
				^a^	^b^			
BMI	Adequate	10255 (69.3)	481 (55.4)	73.7	69.8	1.81	1.58-2.08	0.00
	Altered	4548 (30.7)	388 (44.6)					
Systolic Blood Pressure	Adequate	14536 (98.2)	833 (95.9)	47.4	38.0	2.10	1.83-2.42	0.00
	Altered	3946 (26.7)	37 7 (43.4)					
Diastolic Blood Pressure	Adequate	9418 (63.6)	444 (51.1)	55.2	53.5	1.67	1.40-1.92	0.00
	Altered	5385 (36.3)	425 (48.9)					
Family history of Type 2 diabetes	Without	6540 (44.2)	368 (42.3)	1.18	1.12	0.97	0.84-1.10	0.29
	With	8263 (55.8)	501 (57.6)					

No association was found with FHD. In addition, the groups with individuals categorized as altered BMI had a 1.8 higher risk of having prediabetes. Moreover, the participants with increased BP, both SBP and DBP, had 2.1 and 1.6 higher odds of prediabetes, respectively, than individuals with normal BP values. Finally, the changes in risk factor prevalence in 2008, 2009, and 2010 are shown in Table 4. The prevalence of prediabetes and diastolic BP levels incremented their prevalence in 2010 but altered BMI and systolic BP prevalence remained stable over the three years.

**Table 4:** Prevalence comparison between the risk factor categories over the years DF: Degree of freedom. A chi-square test was performed ^a^ Person's Chi-square value, ^b^ Likelihood ratio value

Risk factor		2008 (n=6014), n (%)	2009 (n=3997), n (%)	2010 (N=5661), n (%)	Chi-square test			
					_a_	_b_	DF	P-value
Body mass index	Adequate	4158 (69.1)	2726 (68.0)	3852 (68.0)	1.8	1.6	2	0.397
	Altered	1856 (30.9)	1271 (31.7)	1809 (31.9)				
Diastolic blood pressure	Adequate	5726 (95.2)	3784 (94.6)	5347 (94.4)	3.5	3.4	2	.167
	Altered	288 (4.7)	213 (5.3)	314 (5.5)				
Systolic blood pressure	Adequate	3908 (64.9)	2580 (64.5)	3374 (59.6)	42.	35.7	2	0.00
	Altered	2106 (35.0)	1417 (35.4)	2287 (40.3)				
Fasting glucose level	Adequate	5770 (95.9)	3834 (95.9)	5199 (91.8)	115.8	92.3	2	0.00
	Altered	244 (4.0)	163 (4.0)	462 (8.1)				

**Figure 1:**
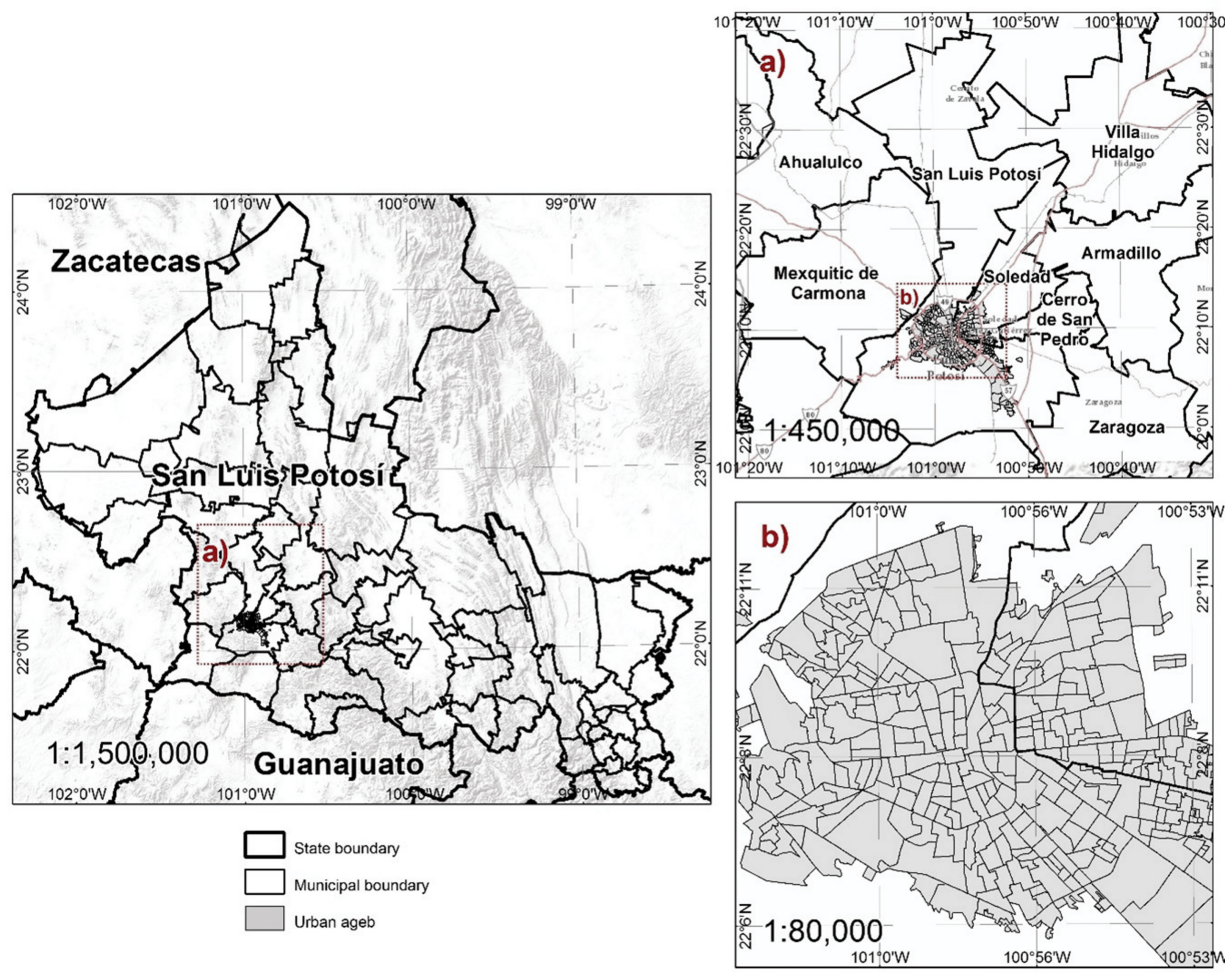
Metropolitan area of San Luis Potosí. Metropolitan area and spatial units of reference (sectors and districts). Image credit: All authors.

The MASLP, as shown in Figure 1, is an urban area consisting of the municipalities of San Luis Potosí, Soledad de Graciano Sanchez, Cerro de San Pedro, Villa de Reyes, and Ahualulco. 

**Figure 2:**
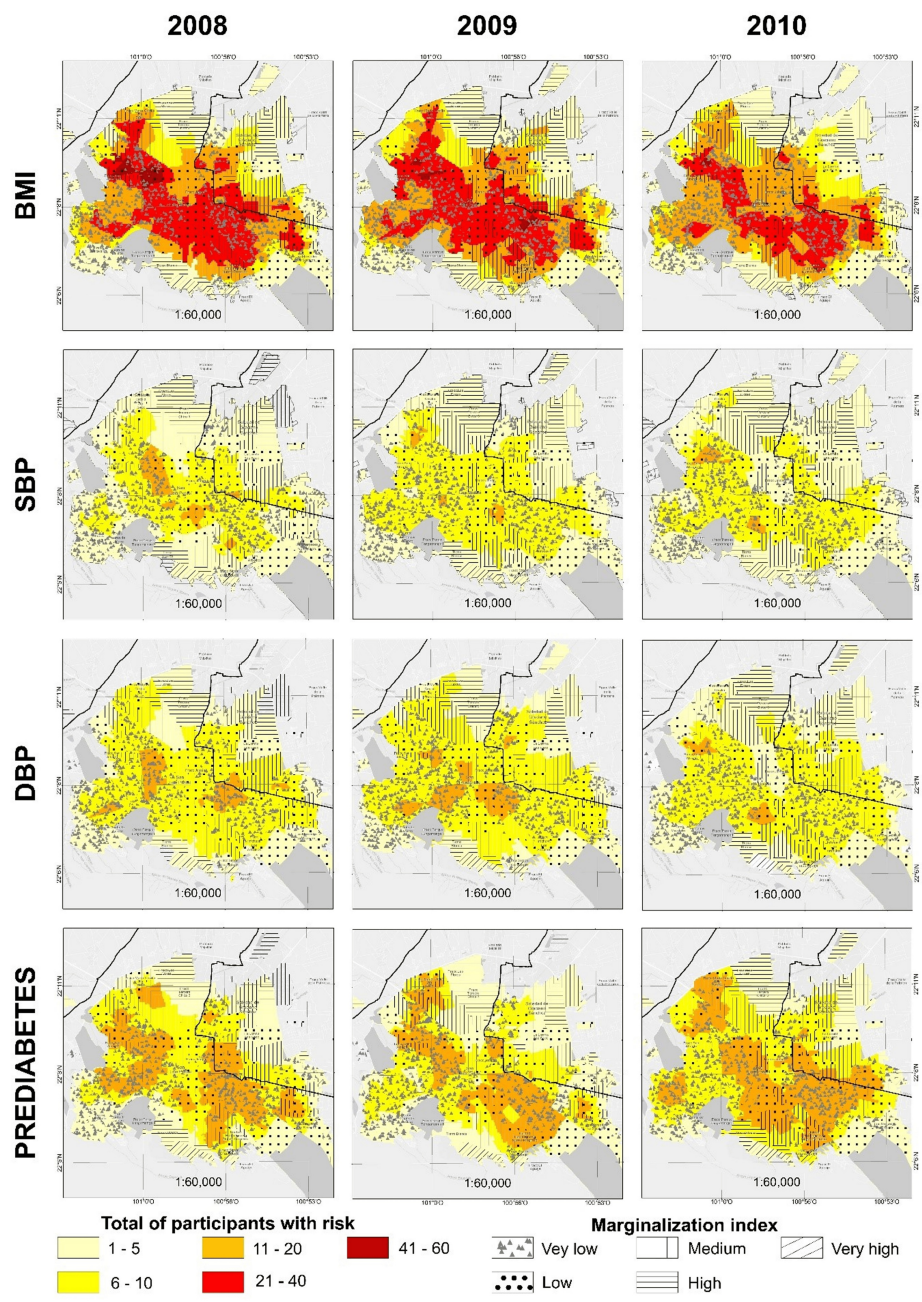
Spatial distribution of the prevalence of prediabetes and associated risk factors of SBP, DBP, and BMI The colors represent clusters of high-risk factor prevalence among late adolescents, according to the marginalization index categories. BMI: body mass index; SBP: systolic blood pressure; DBP: diastolic blood pressure Image credit: All authors

About 90% of the MASLP belongs to San Luis Potosí and Soledad de Graciano Sanchez, where the study was conducted. As of 2020, it has a population of 1,243,980 inhabitants, with an IDG of 0.921. We analyzed the spatial distribution of the variables' prevalence for 2008, 2009, and 2010, only considering the 2010 population for the cartography., with an approximate population of 1,097,906 inhabitants. Figure 2 shows the distribution of BMI, SBP, DBP, and prediabetes in 2008, 2009, and 2010.

Although the number of participants at risk for increased SBP and DBP is lower than those at risk for prediabetes; they are still below the total number of participants at risk for high BMI. Urban AGEBs with 11-20 participants at risk for increased SBP and DBP were in marginalized areas. Nonetheless, these numbers decreased for those at risk for increased SBP in 2009, and this trend was maintained until 2010. The urban AGEBs with 11-20 participants at risk for altered DBP were maintained in 2009 with respect to 2008 but decreased by 2010; thus, the majority of urban AGEBs with 1-10 participants at risk were for elevated DBP.

The urban AGEBs with 1-5 participants at risk for prediabetes in 2008 were located at the periphery of the MASLP. We observed that the AGEB with the highest prevalence of participants (11-20) with prediabetes were mainly concentrated in the areas of medium and low marginalization throughout all years. In 2010, however, there were fewer AGEB with 1-5 participants at risk for prediabetes, and these were located in the northern periphery of the MASLP, where the marginalization index is high, in the southwestern periphery, where the marginalization index is very low, and in the eastern periphery, where the marginalization index is low.

In contrast, the urban AGEB with 6-10 participants at high risk for prediabetes in 2008 was found in three zones: west, southeast, and central to the MASLP, in areas characterized by very low, low, and medium marginalization indexes. In 2009, these urban AGEBs decreased, specifically in the northern part of the MASLP, and by 2010, there was an increase in urban AGEB, with 6-10 participants at high risk for prediabetes, especially in the western part of the MASLP. The marginalization indexes of these areas vary, but the majority of those are categorized as very low and low.

The urban AGEBs with 11-20 participants at high risk for prediabetes in 2008 are located in the western and southeastern part of the MASLP. In 2009, the groups located to the west extended to the north of the MASLP, while the group from the southeast formed a rectangular area. However, in this area, it is evident that the urban AGEBs with 11-20 participants at high risk for prediabetes grew, expanding from the southeast en route to the southwest.

Lastly, the variable with the largest number of participants at risk is BMI, and it is distributed distinctly in the MASLP compared to the other three aforementioned variables. The urban AGEBs with 1-5 participants at risk for elevated BMI are found in the periphery, in areas with high and very high marginalization in the north and south, and with very low and low marginalization indexes in the southwest and east regions. According to our three-year study, the urban AGEBs with 6-10 participants at risk of BMI were located in areas of low and medium marginalization, while urban AGEBs with 11-10 participants at risk of elevated BMI were located in areas with very low, low, and medium marginalization indexes in 2008, 2009, and 2010, respectively. In 2008, urban AGEB with 21-40 at-risk participants were grouped in the south and southeast of the MASLP towards the west, with continuation to the northwest. This pattern continued until 2009 and, although the urban AGEB decreased in 2010, the overall trend remained.

## Discussion

The present study is the first to evaluate the spatial distribution of the prevalence of prediabetes and associated risk factors in a metropolitan city in Mexico using fasting plasma glucose as the definition of prediabetes in a late adolescent context over a three-year span. In 2009, the Cardiovascular Risk Factor Multiple Evaluation in Latin America (CARMELA) Study Investigators reported that from seven Latin American cities, the highest prevalence of prediabetes (3%) was reported in Mexico City, Mexico [21]. Researchers outside of Mexico assessed prediabetes prevalence in young adults and adolescents, finding values of 6.3% and 2.6% in Kuwait and Canada, respectively [22,23]. These values are in contrast with our findings; this may be due to the fact that these observational studies included young adults (aged 18,19,21-26) and other diagnostic criteria for prediabetes were used. Also, the prevalence of prediabetes in male and female university undergraduate students from the south of Mexico was reported with rates of 6.3% and 12.4%, respectively [24]. Another group showed that 10% and 1.5% of undergraduate students from Southern Mexico had prediabetes and T2D, respectively [11]. Moreover, an increased tendency of prediabetes rates was observed over the years, indicating that screening tests in Mexico should be taken more frequently and at younger ages to have a timely differential diagnosis. One study assessed prediabetes prevalence in 2011 and 2016 in Mexican undergraduate students showing a rise in prevalence from 2011 to 2016, similar to our study [25].

It is a fact that T2D and its comorbidities continue to rise. According to our results, the young Mexican population is not an exception. We no longer have the option to treat disease symptoms; we must identify risk factors at a young age to prevent or delay these diseases. In asymptomatic youth, the American Diabetes Association and the American Academy of Pediatrics recommend screening for T2D and prediabetes [26]. However, the United States Preventive Services Task Force (USPSTF) concludes that the evidence is insufficient to evaluate the benefits and harms of T2D screening, and recommends initiating screening tests at 18 years of age [27]. Furthermore, it would be beneficial to comprehend the changes in behaviors over time that could potentially trigger the development of these diseases.

One of our study's strengths was that no other large-scale study had assessed the spatial distribution of prediabetes and associated risk factors in the late adolescent population to date. This highlights the relevance of this study which could contribute to the improvement in population surveillance. Similar studies also found these spatial groupings related to prediabetes, albeit at different scales. For instance, researchers conducted one study in distinct counties in the United States [28], and another in various municipalities across India [14]. In another local study conducted in the Netherlands, no socioeconomic clustering was shown (i.e., prevalence of diabetes) [13]. This was primarily due to the spatially randomized participants' age within the study area, resulting in a decrease in the overall grouping of diabetes prevalence in older people (> 45 years). These results could indicate that although the diabetes clusters identified amongst younger participants were comparable to those in individuals older than 45 years, other reasons unaccounted for and beyond the scope of this study could have played a role.

In contrast with our results, it has been reported that high mortality rates related to diabetes [24] and diabetes diagnosis [15] are related to lower socioeconomic status. A plausible explanation for this difference could be found in the spatial scaling implemented as our study was carried out on a scale with greater granularity than the United States counties. Additionally, those studies used other variables not considered in our study, including the density of fast-food restaurants, the lack of access to grocery stores (i.e., food deserts), the incidence of physical activity (i.e., low physical activity), and the proportion of Hispanic and/or African American individuals [15,24]. This demonstrates that improvements in socioeconomic status and access to healthy foods may significantly reduce the rates of people with diabetes in United States counties.

The correlation between prediabetes and other risk factors, like altered or increased BMI values, implies that we can customize interventions based on the prevalence of these associated factors in specific regions [13]. We discovered that areas with very low, low, and medium marginalization indexes for the MASLP host the largest number of urban AGEB participants at risk of overweight and obesity. A recent study in Indonesia, identified 20 childhood overweight and 36 obesity clusters [29], thereby clearly defining the specific concentration of this phenomenon. Alkerwi et al. report non-significant spatial patterns regarding smoking, diabetes, total serum cholesterol, and low glomerular filtration rate risk distribution in Luxembourg [30]. It is plausible that a lack of significance in spatial patterning between diabetes and these variables is owed to the relatively homogeneous population in that country. Thus, a potential hypothesis is that in an environmentally and socio-economically heterogeneous space, these may generate clustered patterns in those spaces with a risk of diabetes prevalence, demonstrating that diabetes is not a random phenomenon but rather reflected in the clustered patterns.

One limitation of our study is that the targeted population was young residents of San Luis Potosí; therefore, it is unknown whether the behavior of the studied variables in other Mexican states and on different age populations will remain congruent. Furthermore, fasting plasma glucose is not the only method to diagnose T2D and prediabetes. HbA1c tests have been recommended by the ADA as a diagnostic test for diabetes and prediabetes in adults and children. The HbA1c test possesses lower variability compared with glucose measures. However, several non-glycemic factors can affect HbA1c levels [6], and the use of HbA1c for prediabetes diagnosis requires an additional test [31]. Therefore, future research should combine FBG and HbA1C testing as an optimal and accurate approach to identifying prediabetes.

## Conclusions

Spatial analysis of prediabetes prevalence can identify local areas at high risk that would require targeted intervention. The prevalence of prediabetes in San Luis Potosi was lower than other metropolitan areas in Mexico. Furthermore, increased BMI and prediabetes were homogeneously distributed in our spatial analysis.

The urban AGEB with the largest number of participants at risk of altered BMI were found in areas with very low, low, and medium marginalization of the MASLP, and these were grouped from the south and southeast toward the west, continuing to the northwest of the MASLP. This pattern was continuously maintained for 2008, 2009, and 2010, indicating that prediabetes is not a spatially random phenomenon. Future research may benefit from a spatial approach explaining or predicting the prevalence of prediabetes in young people at a local scale.
